# Supplementary material for: A multi-layer mean-field model of the cerebellum embedding microstructure and population-specific dynamics
Source: PLoS Comput Biol. 2023 Sep 1;19(9):e1011434. doi: 10.1371/journal.pcbi.1011434 (PMC10501640; doi:10.1371/journal.pcbi.1011434)
Supplement: S1 Table — Parameters specific of the type of neurons included in the multi-layer MF populations. The parameters in the top part are chosen according to literature, while the parameters at the bottom were extracted from spiking neural network simulating the cerebellar cortex spiking activity. mf = mossy fibers, GrC = Granule Cells, GoC = Golgi Cells, MLI = Molecular Layer Interneurons (Basket cells and Stellate cells) (DOCX) [file pcbi.1011434.s001.docx]

S1 Table. Neuron parameters

| **Parameter** | **Name** | **unit** | **GrC** | **GoC** | **MLI** | **PC** |
| --- | --- | --- | --- | --- | --- | --- |
| **g_L_** | Leak conductance | nS | 0.29 | 3.30 | 1.60 | 7.10 |
| **C_m_** | Membrane capacitance | pF | 7.00 | 145.00 | 14.60 | 334.00 |
| **𝜏_ref_** | Refractory time | ns | 1.50 | 2.00 | 1.59 | 0.50 |
| **𝜏_m_** | Membrane time constant | ns | 24.15 | 44.00 | 9.12 | 47.00 |
| **E_L_** | Resting potential | mV | -62.00 | -62.00 | -68.00 | -59.00 |
| **V_th_** | Threshold potential | mV | -41.00 | -55.00 | -53.00 | -43.00 |
| **V_r_** | Reset potential | mV | -70.00 | -75.00 | -78.00 | -69.00 |
| **k_adap_** | Adaptation constant | MH^-1^ | 0.02 | 0.22 | 2.03 | 1.50 |
| **k2** | Adaptation constant | ms^-1^ | 0.04 | 0.02 | 1.10 | 0.04 |
| **k1** | Decay rate | ms^-1^ | 0.31 | 0.03 | 1.89 | 0.19 |
| **A2** | Update constant | pA | -0.94 | 170.01 | 5.86 | 172.62 |
| **A1** | Update constant | pA | 0.01 | 259.99 | 5.95 | 157.62 |
| **I_e_** | Endogenous current | pA | -0.89 | 16.21 | 4.45 | 891.04 |

Parameters specific of the type of neurons included in the multi-layer MF populations. The parameters in the top part are chosen according to literature , while the parameters at the bottom were extracted from spiking neural network simulating the cerebellar cortex spiking activity. mf = mossy fibers, GrC = Granule Cells, GoC = Golgi Cells, MLI = Molecular Layer Interneurons (Basket cells and Stellate cells)
