# Supplementary material for: A multi-layer mean-field model of the cerebellum embedding microstructure and population-specific dynamics
Source: PLoS Comput Biol. 2023 Sep 1;19(9):e1011434. doi: 10.1371/journal.pcbi.1011434 (PMC10501640; doi:10.1371/journal.pcbi.1011434)
Supplement: S2 Table — Parameters used to set up the inter-population connectivity of the multi-layer MF cerebellar network. Parameters were extracted from the spiking neural network simulating the cerebellar cortex spiking activity. mf = mossy fibers, GrC = Granule Cells, GoC = Golgi Cells, MLI = Molecular Layer Interneurons (Basket cells and Stellate cells). K = pre-synaptic connectivity resulting by weighting the mean synaptic convergence with the number of synapses; Q = quantal synaptic conductance in nS; τ = synaptic decay time constant; Erev = reversal potential that is 0 V for excitatory synaptic connections and -80 V for inhibitory synaptic connections. (DOCX) [file pcbi.1011434.s002.docx]

S2 Table. Presynaptic parameters

| **Presynaptic connection** | **K** | **Q** [nS] | **𝜏** [ms] | **E_rev_** [V] |
| --- | --- | --- | --- | --- |
| **mf-GrC** | 4.00 | 0.230 | 1.9 | 0 |
| **GoC-GrC** | 3.50 | 0.240 | 4.5 | -80 |
| **mf-GoC** | 57.10 | 0.240 | 5.0 | 0 |
| **GrC-GoC** | 501.98 | 0.437 | 1.25 | 0 |
| **GoC-GoC** | 2592.00 | 0.007 | 5.0 | -80 |
| **GrC-MLI** | 243.96 | 0.154 | 0.64 | 0 |
| **MLI-MLI** | 1418.69 | 0.005 | 2.0 | -80 |
| **GrC-PC** | 489.16 | 0.510 | 1.1 | 0 |
| **MLI-PC** | 10.28 | 1.244 | 2.8 | -80 |

Parameters used to set up the inter-population connectivity of the multi-layer MF cerebellar network. Parameters were extracted from the spiking neural network simulating the cerebellar cortex spiking activity. mf = mossy fibers, GrC = Granule Cells, GoC = Golgi Cells, MLI = Molecular Layer Interneurons (Basket cells and Stellate cells). K = pre-synaptic connectivity resulting by weighting the mean synaptic convergence with the number of synapses; Q = quantal synaptic conductance in nS; 𝜏 = synaptic decay time constant; E_rev_ = reversal potential that is 0 V for excitatory synaptic connections and -80 V for inhibitory synaptic connections.
