# Supplementary material for: A multi-layer mean-field model of the cerebellum embedding microstructure and population-specific dynamics
Source: PLoS Comput Biol. 2023 Sep 1;19(9):e1011434. doi: 10.1371/journal.pcbi.1011434 (PMC10501640; doi:10.1371/journal.pcbi.1011434)
Supplement: S3 Table — (DOCX) [file pcbi.1011434.s003.docx]

S3 Table. Mean-field symbols

| **Symbol** | Description |
| --- | --- |
| **AdEx** | Adaptive Exponential (model) |
| **BC** | Basket Cells |
| **BSB** | Brain Scaffold Builder |
| **c** | Covariance between populations activity |
| **DCN** | Deep Cerebellar Nucleus |
| **E-GLIF** | Extended Generalized Leaky Integrate and Fire (model) |
| **F** | Transfer Function [Hz] |
| **erfc** | Error function |
| **GoC** | Golgi Cells |
| **GrC** | Granule Cells |
| **K** | Mean synaptic convergence |
| **LC** | Lugaro Cells |
| **LFP** | Local Field Potential |
| **MF** | Mean Field |
| **MLI** | Molecular Layer Interneurons |
| **μ_G_** | Population-specific conductance [S] |
| **μv** | Average of membrane potential fluctuations [V] |
| **N** | Cells number in the population |
| **PC** | Purkinje Cells |
| **pf** | parallel fibers |
| **Q** | Quantal synaptic conductance [S] |
| **SC** | Stellate Cells |
| **SNN** | Spiking Neural Network |
| **σ_V_** | Standard deviation of membrane potential fluctuations [V] |
| **T** | Mean Field Time constant [s] |
| **τ** | Synaptic time decay constant [s] |
| **τ_V_** | Autocorrelation time of membrane potential fluctuations [s] |
| **UBC** | Unipolar Brush Cells |
| **ν** | population activity [Hz] |
| **ν_drive_** | Driving input [Hz] |
| **V^eff^_thre_** | Phenomenological Threshold [V] |
|  |  |

Populations of mean-field model and parameters included in the pipeline.
