# Supplementary figures and images for: A multi-layer mean-field model of the cerebellum embedding microstructure and population-specific dynamics
Source: PLoS Comput Biol. 2023 Sep 1;19(9):e1011434. doi: 10.1371/journal.pcbi.1011434 (PMC10501640; doi:10.1371/journal.pcbi.1011434)

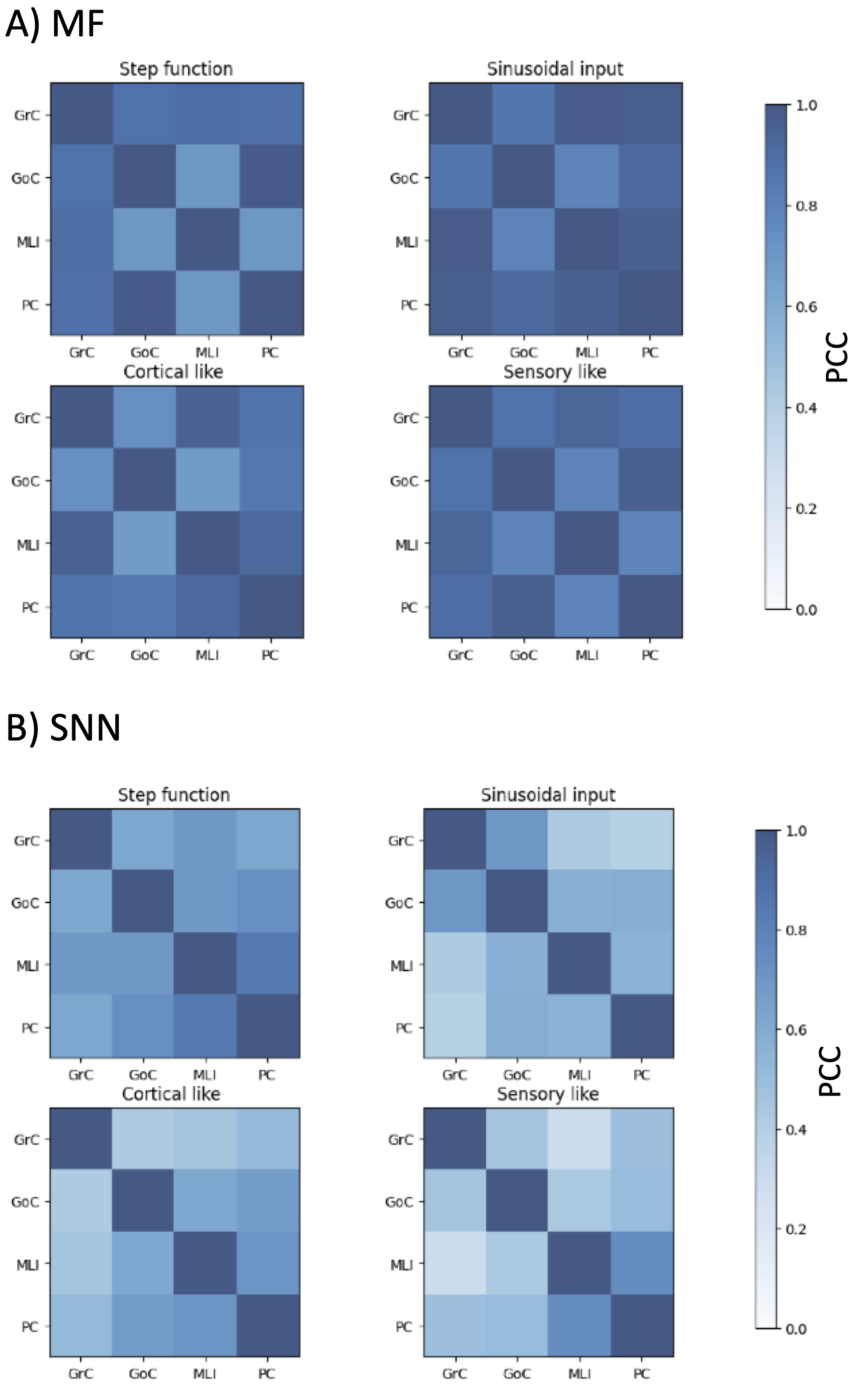

Supplement: S1 Fig — Population activities predicted by cerebellar MF were highly cross-correlated, resulting in Pearson Correlation Coefficients always > 0.7 (Panel A). Correlation between population activities simulated by SNN is reported in Panel B; variability intrinsic in SNN led to lower correlations between pairs of population activities than in MF. These correlations matrices are reported for each input pattern. The input frequencies might be replaced with a probabilistic kernel to introduce variability in the MF formalism and reduce the inter-population correlations. (TIFF) [file pcbi.1011434.s004.tiff]
