# Supplementary material for: A multi-layer mean-field model of the cerebellum embedding microstructure and population-specific dynamics
Source: PLoS Comput Biol. 2023 Sep 1;19(9):e1011434. doi: 10.1371/journal.pcbi.1011434 (PMC10501640; doi:10.1371/journal.pcbi.1011434)
Supplement: Attachment — Submitted filename: PCOMPBIOL-D-22-01885_review.pdf [file pcbi.1011434.s005.pdf]

The authors develop and validate a mean-field model of the cerebellar microcircuit with focus on biological grounding. For this, they chose an approach employed previously to derive mean-field models of cortical circuits, where each neuronal population is represented in terms of statistical moments of activity (firing rate), and a transfer functions which computes the average population activity as a function of presynaptic population activity.

In particular, the work presented here builds on previously published spiking neural network (SNN) model of the cerebellar microcircuit composed of four interconnected populations. The SNN model serves both as a template for the architecture of the mean field model in terms of involved neuronal populations and their connectivity, and to simulate data for fitting the transfer functions for individual populations. The resulting mean-field model has 20 state variables including population activities, driving input, and respective covariances (eq. 13). Lastly, the time-constant of the mean-field model was set to optimize the model predictions with respect to the experimental LFP recordings of acute mouse cerebellar slices.

The validation of the model is thorough and convincing, and is based of two parts. First, the response of the mean-field model to four types of stimulus is compared to the response of the equivalent SNN. The reported results in Figure 5 are clear, however it would be also useful to compare the second moments of the individual neuronal populations between the mean-field model and the SNN - e.g. as a supplementary figure. Second, the response to a step function stimulus is evaluated when the selected connections between the populations are modulated to mimic effects of inhibitory control and long term plasticity known in literature. Here, however, it should be clarified how the modulation of parallel fibers w_{pf-PC} was implemented: the parallel fibers are not defined in the model derivation - I'd assume it the $GrC \to PC$ connection. It should be marked in the diagram of Figure 2.

I have questions regarding the section 4.3 on performance. The authors claim that the mean-field reduced the computation time by 60%, which is far too little for reduction from a simulation of $\approx 3x10^4$ neurons to 20 differential equations. Additionally, the authors claim that this should be further improved by replacing the TF fitting procedure with a look-up table. Does that mean that the authors are performing the fitting procedure for each run of the mean field model? If so, why?

The mouse LFP data was used to determine an important time-constant parameter of the model, and also the SNN was developed and validated with respect to mouse empirical data. The authors should be more explicit about this and discuss the challenges of translating the work to human brain models.

Beside that I would have following specific comments:

- There are other approaches to Mean Field modeling than those based on Transfer Functions, some should be mentioned in the introduction. For example:
  - Schwalger, T., Deger, M., & Gerstner, W. (2017). Towards a theory of cortical columns: From spiking neurons to interacting neural populations of finite size. *PLoS Computational Biology*, *13*(4), e1005507.

    - Coombes, S., & Byrne, Á. (2019). Next generation neural mass models. In *Nonlinear Dynamics in Computational Neuroscience* (pp. 1–16). Springer International Publishing.
    - Montbrió, E., Pazó, D., & Roxin, A. (2015). Macroscopic Description for Networks of Spiking Neurons. *Physical Review X*, *5*(2), 021028.
- The linked code is not a public repository
- The text needs to be checked for typos, for example: line 61 - behaviur; line 482 - Figure 3.5
