# Supplementary material for: A multi-layer mean-field model of the cerebellum embedding microstructure and population-specific dynamics
Source: PLoS Comput Biol. 2023 Sep 1;19(9):e1011434. doi: 10.1371/journal.pcbi.1011434 (PMC10501640; doi:10.1371/journal.pcbi.1011434)
Supplement: Attachment — Submitted filename: Responses_to_review.docx [file pcbi.1011434.s006.docx]

Dear Editors,

Please find the revised manuscript entitled **“A multi-layer mean-field model of the cerebellum embedding microstructure and population-specific dynamics**”. We submit a manuscript with an updated introduction now including now overview of different MF approaches used in neuroscience to further clarify the context of the approach applied here. We performed further analysis to provide a deeper insight on our model dynamics and to provide a rigorous comparison with the spiking neural network. We revised the discussion adding practical applicability example of our model and we add section *4.4. Limitations and further developments* to deepen the comparison with the SNN. Further, we are pleased to provide here the detailed list of our responses to the review comments.

On behalf of the authors,

Roberta Lorenzi.

## **Responses to the reviewers**

## Reviewer #1

We thank Reviewer 1 for his detailed review and constructive comments.

*This manuscript translates a highly detailed SNN model of the cerebellum into a significantly simpler mean-field model.* The manuscript is very relevant and the research therein has great value**.** *According to the authors this is the first time this is done for the cerebellum, which is correct to my knowledge. The many ways in which biology underlies the SNN model are very compelling, particularly microanatomy, biophysics, and the translation into an MF model is valuable from the context of whole brain models. A MF model of the cerebellum has great value and potential in explaining mesoscopic behavior of electric fields and behavior of populations under a variety of conditions.

However, the shortcomings of a model are as interesting as its power and merit in -depth analysis of the deviations between the SNN and MF. Rather than repeatedly bolstering the value of the type of modeling, the authors should focus on systematically evaluating the cases in which the model is applicable. There are a few sentences that aim at this objective, but IMHO they often offer shallow reasoning. Similarly, both introduction and discussion lack depth.*

Based on Reviewer #1 suggestions, we have now included in the introduction an overview of different MF approaches (Lorenzian functions and finite-element based) used in neuroscience to further clarify the context of the approach applied here:

*The mean-field (MF) approximation theory describes collective neural activities maintaining a direct link with the corresponding spiking neural network. The MF model formalism approximates high-dimensional random systems by averaging their original properties over degrees of freedom to compute the first two statistical moments of the system variables (e.g., mean and variance of the population firing rates in a neural network). Different mathematical approaches exist to derive such statistical descriptions depending on the complexity of the underlying network. While theoretical derivations in simpler settings allow to capture firing dynamics up to fast synchronized events (e.g., Lorentzian or finite-size effect-based formalisms) (10-13), the network considered here impedes such an approach. Instead, we use a formalism based on the definition of a transfer function (TF) that models the neuronal input-output relationship to investigate the properties of network responses to different input (7,10,11). The MF developed here is based on the TF approach that summarizes the neuronal and connectivity properties of an entire SNN through ad-hoc transfer functions (11–13) and captures the statistical properties of network activity by computing the probabilistic evolution of neuronal states at subsequent time intervals (10,14,15).*

Further, in the discussion we have added section *4.4 “Limitations and further developments”* in order to deepen the comparison with the SNN. In summary we pointed out the general shortcomings of MFs compared to the SNN. Specifically, with MF approaches the granularity level becomes coarser, resulting in a limitation in studying the role of a single neuron. Indeed, MFs are models at the scale of neural populations, thus they can't reproduce all mechanisms deriving from neuronal heterogeneity within the same population. Moreover, MFs are rated-coded machines, while SNN are timing machines and in some cases this could lead to a timing issue.

We further discuss the predictive power of our model which catches complex dynamics like burst-pause of Purkinje Cells (PC), but it can't reproduce mechanisms like population oscillations or bursts driven by synchronization among single neurons ([Sedaghat-Nejad](https://www.pnas.org/doi/10.1073/pnas.2118954119#con1) et al., 2022) and it doesn’t include PC plasticity as SNN does. We introduced this in section *4.2 MF prediction : “Although the cerebellar MF was able to replicate changes in activity patterns characterizing learning states, it was not possible yet to simulate the long-term plasticity rules that would be needed to autonomously generate the PC learning curves”*

and, within this context, in section *4.4 “Limitations and further developments”* we introduced a further development of our model to add synaptic plasticity remaining at the mesoscale: *“Another improvement of the cerebellar MF could include synaptic plasticity rules like in the corresponding SNN (105). Such mechanisms at a mesoscopic scale could be modeled by implementing changes of synaptic strength based on the firing rates”*

We have expanded the discussion (section 4.2) about the applicability of the model, pointing out that it could be used as a bridge to translate microscale properties into upper scale. *“As an example, identifying and extracting biophysical meaningful features from atlases, such as the receptor maps (e.g., extracted form Allen Brain Atlas, http://ww.brain-map.org), might allow combining mesoscopic MF simulation with characteristics of molecular functions.”*

We have also added practical example about how to integrate this models into macroscale simulators such as The Virtual Brain (TVB) to investigate signal propagations in the cerebro cerebellar loop: *“Different modules of cerebellar MFs could be connected one to another and with other cortical and subcortical MFs following subject-specific connectome rules that could be extracted from imaging techniques such as connectivity matrices computed from diffusion weighted imaging (124).”*

Moreover, we have performed a further analysis to investigate the Input/output relationship of our mean field and we added *Figure 5) Cerebellar mean-field overall input/output relationship.* We also computed correlation matrices between population activities, in MF and SNN networks (Figure S1 in supplementary material).

*It seems to me that the manuscript finds it difficult to find a balance between the simplifications necessary for the MF model and its explanatory power. There are multiple statements trying to justify its use and exagerating its capabilities. At times it seems to me that the authors are not fully compelled by their own model and hope that by repeating the claims, these are justified. Perhaps if the manuscript is improved to cover some of those use cases, the authors would not need to go to such lengths to argue their approach. In fact, it does not appear to me that MF models need to be justified at all, they have already shown their value in a large number of publications over the last 40 years. The manuscript should maintain a more critical and factual stance, focusing on objective description and curtailing the sales pitch. Authors should prefer a neutral language throughout the manuscript.*

We have revised the language throughout the manuscript, as suggested, and we provide some practical examples of use-case and applicability of the model both for general cases (please, refer to the answers above), and specifically for some pathologies (answers below).

*For instance, the manuscript belabors the point that the MF model is of great biophysical relevance (e.g. section 4.3), but the discussions remains abstract and do not sufficiently establish the scope of use beyond multiple mentions to whole brain modeling / pathology. Clearly some pathologies are betters studied via spiking models. Please discuss with more clarity: When should we consider an MF rather than a spiking model?*

Now, in the discussion, we disangle the scope of MF and SNN. We would like to explicitly claim that our MF is not intended to replace a SNN but it’s an alternative tool that might be considered and we report two examples to further clarify how MF and SNN could be employed to study different aspects of pathologies :

*“Theoretically, the cerebellar MF is not just a replacement for SNN, but rather a specific instrument for addressing questions at a mesoscale. SNNs and MFs indeed cover complementary aspects of brain function and dysfunction. For example, in Parkinson disease, a SNN was successfully used to simulate the thalamic and basal ganglia microcircuits in order to control deep brain stimulation electrodes (95,96). The SNN provided a description of microcircuit activity and of the involvement of dopaminergic neurons in tremor (97,98). On the other hand, MFs of the basal ganglia and thalamocortical circuit were used to embed neural mechanisms into the motor network involved in Parkinson disease and investigate large-scale oscillations (99). In dystonia, a cerebellar SNN embedding Dystonic alterations was successfully used to simulate alterations in PC firing rate , olivocerebellar connectivity, and parallel-fiber/PC spike-timing dependent plasticity (STDP) (100). While these microscopic properties may not be reproduced by the cerebellar MF, it may however reproduce the overall PC output alterations allowing to simulate the large-scale propagation of cerebellar abnormalities to other brain regions.”*

Further, in section 4.4, we clarify with a practical example a case in which the time-coding performed by SNN can’t be reproduced by a MF formalism:

*“For example, a cerebellar SNN was used to simulate the control of saccades movements exerted by two subpopulation of PCs (104). Conversely, the cerebellar MF assumes intra-population homogeneity and can’t be used to study such phenomena”*

*In terms of scope and limitations multiple aspects of the translation from SNN to MF merit deeper discussion. Clearly, in terms of explaining cerebellar computations, a continuous system such as this fails to capture many relevant aspects of cerebellar operation. For example:*

We have updated the manuscript with the new section *4.4. Limitations and further developments* to clarify that MF is designed to reduce the dimensionality of the system and the tradeoff of simplification is the loss of some properties that the original system has. These include topology and spatial topography as well spike coding in the time domain (here rate coding). We also would like to provide specific answers for the specific comments below.

*- CF related synchronous pauses in PN are impossible*

The burst-pause pattern is captured by the cerebella MF. If the cf would be in the model, this would presumably generate the burst-pause response. Inserting the cf as an additional PC input would require a new TF for the PC*.*

*- Influence of local microcircuit topology AND topography (e.g. directionality of PN collaterals)*

Clearly, the MF is a self-consisting module: if the connections are intended internally, then the MF could be rewritten with modified TFs. Otherwise, is the connections are intended externally, this could be accomplished by wiring multiple MFs with specific topology and topography within the brain connectome.

*??- Can it be used to represent the surfaces of the neuropils (can it become a PDE)?*

In principle yes, if the MFM represents a module small enough to be representative of the underlying neuropils. Large-scale surface can be represented by interconnecting different modules of MF and describing the signal propagation with traveling waves

*??- Does low dimensional representation of spikes preclude representation of high dimensional sensory inputs?*

If high-dimensional sensory inputs are encoded at the population level with different subgroups of the population encoding a specific feature or a different combination of features, it could be a limitation. On the other hand, if a high-dimensional sensory input is elaborate at population level this shouldn’t be a limitation. Further, with many interconnected modules it would be possible to simulate the activity of the cerebellar cortex in response to input coming from the other regions. The number of the modules and the complexity of the sensory input will depend on the granularity of the connectome data.

*- How to study reorganization at cell level?*

In case of alteration at population level, it is possible to study this condition by modifying the parameters of synaptic connectivity, specifically the synaptic mean convergence, the synaptic time decay and the quantal conductance, according to the condition of interest.

*What about coupling at different temporal and spatial scales (gap junctions / glutamate spillover)*
Both in the spiking network that works as a template, but not explicitly in the MFM. Therefore both parameters are accounted for through optimization against the SNN template. In detail, for the gap junction it is not possible to represent explicitly within the same cell population (which is already collapsed). Regarding glutamate spillover, it generates a different temporal scale that would require a second synapse and spatial scale that is not possible to directly account for.

*- What about the significantly reduced variability of responses in comparison to the SNN*

In future developments, variability could be introduced into the MFM by adding probabilistic kernels to certain parameters following the approach used in *Di Volo M, Destexhe A, Sci Rep(2021).*

*??- Does the size of the population being represented influences the time constants of the model?*

By changing any one of the four MF populations, we may expect that time constant changes. As an example, in the case of a change of a specific population size due to an altered condition we expect a change in the time constant with a consequently change in the predicted dynamics

*- How about oscillations in the intrinsically active SNN populations? Are there any, or can we exclude this from the model?*

From a microscopic point of view, in our bottom-up reconstruction we have maintained salient properties of neurons, including intrinsic neuronal oscillations. The basal firing frequency, indeed, is a point in the neuron I-O function. By changing it, we expect the MF model I-O function also changes*.*

Further, from a mesoscopic perspective, the formalism of our cerebellar MF is intended to work at asynchronous state with irregular firing rate. Therefore, in section *4.4 Limitations and further developments* we clarified that input oscillations are not processed by this formalism. Oscillations in Figure 6 are merely input-driven. To include frequency elaboration as an intrinsic process of the cerebellar MF itself, further improvements in the MF formalism should be introduced, e.g. adaptation and heterogeneity.

*Other Comments:*

*The listed motivation for developing the MF model is that a brain region specific MF is better than a generic, existing MF because these “[...] do not capture in full the complex properties of specific neuronal populations [...]”. This is never quantified, and we never see an actual comparison to such a generic MF model - leaving the question, does it actually perform better than the generic model? The justification to develop the model is purely based on the availability of data (line 106). Yet, in the discussion, it is stated that “However, the cerebellar cortex MF benefitted of a previously validated SNN [..]” - this statement is not backed up and I’m not yet convinced that this is an improvement over a generic MF model (I mean personally I might be convinced).*

“Generic” MF models, like the one we used as a reference (Zerlaut et al., 2018) or the Wong and Wang, 2006, proved to be reliable for the description of cortical dynamics. However , the structural and functional differentiation of subcortical regions prompts for the development of specific MF models. We further discuss this aspect in *section 4.2: “In contrast to conventional MFs, which are often a black box in terms of physiological parametrization, the cerebellar MF internal model parameters inform the user about the average properties and variance of fundamental mechanisms in the circuit (e.g., intrinsic and synaptic excitation) and can therefore be used to remap biological properties”.*

Concerning the cerebellum, in a previous work (Palesi et al., 2020) we showed that the Wong-Wang model provides an initial approximation of local cerebellar dynamics. However, the W-W model does not capture several properties that are deemed to be critical for cerebellar computation, e.g., the interneuron network and the inhibitory output from Purkinje neurons. Further, this parallelism with biophysical parameters allows flexible manipulations to reproduce specific cerebellar pathologies. Therefore, region-specific models like ours are designed to improve simulations of whole-brain dynamics. The fact that we have a previously validated SNN is a definite advantage, since it provides the backbone for MF reconstruction and the dataset for MF model validation. *Chosen model complexity: A semi-analytical TF is used to define the MF. This TF is started as a template. Where did this specific template come from? Why aren’t there more/less terms in the model template (seems like the alpha term is explained partly in line 600 in the discussion)? Were other models considered?*

The semi-analytical approach to derive the MF model is motivated by the complexity of the SNN considered here. No other models were considered because, to our knowledge, there is no other approach applicable to the neuronal and synaptic properties of our SNN. Neither (1) computing the TF by deriving the first passage time using a Fokker-Planck approach (as in Amit & Brunel, Cereb. Cortex 1997) nor (2) deriving the time-varying density function (as in Montbrio et al., PRE 2015) are analytically tractable approaches in this case.
Concerning the number of terms, our previous study (Zerlaut et al., J. Physiol. 2016) reports that this number of terms was found as a good compromise between the flexibility of the fit (capturing the TF of qualitatively different neuronal models), the robustness of the fit and a limited number of terms. We did try to add more terms to improve the goodness-to-fit in the TF (from a first order to a second order multivariate polynomial in the effective threshold, as in Zerlaut et al., JCNS 2018), however the fit was found to be unreliable due to the large input space characterizing our cerebellar model (Fig. 3). Therefore we stick to the reference to our original study and we just report the changes that were introduced: the introduction of the *alpha* term (as already done in ref. 21) that is now further explained in the updated main text:
*Since the erfc is a sigmoidal-shaped function limited between -1 and 1, it did not accurately follow the numerical TF distribution at the boundaries, losing precision at high frequencies. This problem was circumvented by tuning a factor, alpha (equation 5), similarly to what was done for isocortical models (21), which does not represent a physiological quantity, but is able to extend the TF reliably over the whole range of physiological input frequencies.*

We now stress the rationale behind this approach (the ability to capture the TF in a relatively complex biophysical setting) in the updated Introduction.

*Limitations (discussion): Among other simplifications, only the 4 main neural populations of the cortex were modeled; how does this show up in the results? In line 636 GoC behavior is discussed, is this the only behavior does this model not capture correctly? In what cases would one be better off using the more detailed SNN?*

We update “MF Tuning” in section 4.1 by clarifying: *“The cerebellar MF reproduced the known aspects of circuit physiology revealed experimentally. These include: the ability of GrCs both to respond to impulsive inputs with bursts curtailed by GoC inhibition and to faithfully follow slow input fluctuations (94); the ability of PCs to generate burst-pause responses accentuated by MLIs (48,51). The cerebellar MF behaved as a smoothing filter reproducing the trend of SNN simulations without capturing the intra-population variability (e.g., GrC fluctuations in response to a constant input - Figure 6A). Since the intra-population variability was not accounted for, the maximal GoC responses were underestimated by the MF, which approximated GoG to a homogeneous population neglecting their biological heterogeneity (88). The differences that could be observed between the MF and the corresponding SNN simulations could thus be explained as a drawback of the homogeneity assumption. Despite these aspects (further discussed in section 4.4) the MF predicted with good approximation the SNN cerebellar output, i.e., the PC activity (Boxplot in Figure 6).”*

The deviations between MF and SNN reflect the difference between having or not having spikes and this difference is now quantified with Root Mean Square Error computation (*“For each stimulation protocol, the PC activity predicted by the MF laid within the variability range of the corresponding SNN (the RMSE between MF and SNN was ~ 30 %)”)*

*Underlying model: Why is the chosen cerebellar cortex SNN the right underlying model to develop the MF model? No comparison to other cerebellar cortex models is made (do these exist?), and why then this exact model is chosen.*

We have chosen the most advanced spiking model available optimized with experimental recordings and multicompartmental simulation (Masoli et al., 2015; Geminiani et al., 2019; De Schepper et al., 2022, Monteverdi et al., 2023).

*Figure 3, Multiple readability issues. Also, in B due to overlapping color schemes the overlap between lines and points is confusing. Legend needs edits for readability.*

We update the caption of Figure 3 to clarify: *“****Figure 3)*** ***Numerical Transfer Functions (TFs) and the corresponding Analytical fitting****. The simulation used to compute the numerical TFs last 5 seconds, with a time step = 0.1 ms.* ***A)*** *A* *2D numerical TF template is reported for Granule Cells (GrC), Molecular Layer Interneurons (MLI) and Purkinje Cells (PC), which receive inputs from two presynaptic populations (x axis: excitatory input; y axis: inhibitory input; color bar: numerical TF color code). A 3D numerical TF template is reported for Golgi Cells (GoC), which receive input from 3 presynaptic sources, i.e., mossy fibers, GrC and GoC (showed on the axis, color bar: GoC numerical TF color code). From the 3D domain of frequencies combination, only the physiological working frequencies of GrC and GoC are considered for mossy fibers inputs from 0 Hz to 80 Hz, as obtained in corresponding spiking neural network simulations and 3D numerical TF for GoC which receive three presynaptic inputs.* ***B)*** *Numerical TFs (points) are used to fit the corresponding analytical TFs (lines, νout on y axis). Excitatory inputs are reported on the x axis, inhibitory ones are color-coded (color bar). The 2D analytical TF presents a non-linear trend, while the GoC Analytical TF shape is almost linear both for lower 𝜈_drive_ = 20 Hz and higher 𝜈_drive_ = 80 Hz.”*

*Figure 5): “[..] the MF is able to respond to the same different stimulation patterns within the same frequency ranges of SNN” -> 1) This is never quantified (should I base this statement on the boxplots being next to each other?). 2) For the step function I wouldn’t necessarily agree that the frequency ranges overlap. What creates the differences here? This deserves careful discussion as it is one of the main differences between SNNs and MF and define the scope of applicability of the MF model.*

Figure 5 is now Figure 6 and the manuscript was changed accordingly to express the differences between MF and SNN which lead to the mismatching in the simulated output (see also above). We clarify that the cerebellar MF actually reproduces the pattern of SNN simulations capturing the trend of the activity with the exception fluctuations driven by the intra-population variance and (e.g., plateau of GrC activity in Figure 6 Panel A) and of the maximal GoC responses. We quantify the mismatch by computing RMSE and correlation matrices by adding a Figure in Supplementary material (Figure S1) and we further discuss the applicability of MF compared to the applicability of SNN (please refer to the answers above).

*Nitpicks:*

We thank the reviewer for noticing, we have fixed the specific points.

*Line 164, equation (1), page 8. Missing brace in the first equation.
Line 230, equation (5) use \mathrm{erfc} or \operator{erfc}
Line 246, equation (6) extraneous dot after equals sign
Line 292, equation (10) missing bracket under the fraction
Line 297, firing ‘behaviors’
Line 332, equation (13) why is there a large bracket on the right side?
Line 335, repeating “Einstein’s notation”. Also, IMO this adds unnecessary opacity, as this is not known to neurobiologists to whom this paper is addressed.
Line 409, listing the synaptic strengths is absolutely not necessary
Line 413, ‘smaller than’ rather than ‘minor’
Line 415, ‘peaks’
Line 286, equation (Es=) no space? Why an = sign in a sentence (also later in the sentence)
Figure 4B): missing X axis (time?)
Line 572: remove “precisely”. Also, attempt to maintain a more neutral language throughout the manuscript.
Line 587, remove “indeed”
Line 592: =vdrive=
Line 598-599 need editing for clarity. What does it mean that the “erfc” is stiff?
Line 603: remove “precise” as those are in the SNN and not in the MF. An approximation of a precise description is still an approximation.
Line 605: instead of ‘higher scale’, write at a ‘mesoscopic scale’ or ‘coarser’ scale.
Line 613: AFAICS, this argument does not clearly follow from the preceding.
Line 628: whether it is a ‘powerful tool’ is yet to be shown. In the absence of that demonstration, this sentence is void.
Line 643: consider ‘dynamics’ instead of ‘functioning’*

## Reviewer #2

We thank Reviewer 2 for his insightful remarks and helpful comments. We have included his/her points into the revised manuscript and are pleased to provide our responses to his/her specific questions.

*The authors develop and validate a mean-field model of the cerebellar microcircuit with focus on biological grounding. For this, they chose an approach employed previously to derive mean-field models of cortical circuits, where each neuronal population is represented in terms of statistical moments of activity (firing rate), and a transfer functions which computes the average population activity as a function of presynaptic population activity.*

*In particular, the work presented here builds on previously published spiking neural network (SNN) model of the cerebellar microcircuit composed of four interconnected populations.*

*The SNN model serves both as a template for the architecture of the mean field model in terms of involved neuronal populations and their connectivity, and to simulate data for fitting the transfer functions for individual populations. The resulting mean-field model has 20 state variables including population activities, driving input, and respective covariances (eq. 13). Lastly, the time-constant of the mean-field model was set to optimize the model predictions with respect to the experimental LFP recordings of acute mouse cerebellar slices.*

*The validation of the model is thorough and convincing, and is based of two parts. First, the response of the mean-field model to four types of stimulus is compared to the response of the equivalent SNN. The reported results in Figure 5 are clear, however it would be also useful to compare the second moments of the individual neuronal populations between the* mean-field model and the SNN - e.g. as a supplementary figure.

Figure 5 is now Figure 6 since we have added a detailed study on emerging I-O relationships across layers to quantify the functional regime of the model ( *Figure 5) Cerebellar mean-field overall input/output relationship).*

In Figure 6, we have add the covariances predicted by MF as a shade over the average activities. Furthermore, in addition to the Boxplots, we quantified the differences between the MF and the SNN by computing the RMSE (*“For each stimulation protocol, the PC activity predicted by the MF laid within the variability range of the corresponding SNN (the RMSE between MF and SNN was ~ 30 %)”*) . We have also further discussed the discrepancy between SNN and MF in section *3.3 Performance vs Realism* and in a new section *4.4 Limitations and further developments.* Moreover, we explore the (co)variances predicted by our MF computing the correlation matrix added as Supplementary Figure (Figure S1) ).

We hope that doubts on this point are solved.

*Second, the response to a step function stimulus is evaluated when the selected connections between the populations are modulated to mimic effects of inhibitory control and long term plasticity known in literature. Here, however, it should be clarified how the modulation of parallel fibers wpf−PC was implemented: the parallel fibers are not defined in the model derivation. I'd assume it is the connection GrC->PC . It should be marked in the diagram of Figure 2*.

We use pf-PC as a notation to refer to parallel fibers - Purkinje cells connection. However since parallel fibers are not explicitly derived in the model, we changed in the manuscript wpf-pc into wgrc-pc and we clarified as follow: *“To simulate the effects of GrC-PC plasticity on PC dynamics, different synaptic strengths (w_GrC-PC_) were explored, including w_GrC-PC_ = 65% that corresponds to the decrease of GrC-PC synaptic strength during motor learning according to animal experiment(71).”*

I have questions regarding section 4.3 on performance. The authors claim that the mean-field reduced the computation time by 60%, which is far too little for reduction from a simulation of neurons to 20 differential equations. Additionally, the authors claim 01x3 ≈4 that this should be further improved by replacing the TF fitting procedure with a look-up table. Does that mean that the authors are performing the fitting procedure for each run of the mean field model? If so, why?

It’s the erfc function that is computationally expensive (evaluated in scipy through polynomial expansion). Making a table of erfc should speed up the procedure, therefore it will be convenient to implement this with the perspective of an integration into platform like The Virtual Brain.

*The mouse LFP data was used to determine an important time-constant parameter of the model, and also the SNN was developed and validated with respect to mouse empirical data. The authors should be more explicit about this and discuss the challenges of translating the work to human brain models.*

We are currently working on this and we would like to thank the reviewer to give us the chance to explain this point better. We add a perspective paragraph in “Conlcusion and perspective” section summarizing what we are doing. *“The cerebellar MF model developed here was validated against mice data and our promising results prompt for its translation to humans. In principle, the fundamental network architecture will remain the same, while parameters of neuron and synaptic models will require specific retuning. Further model validations should be implemented on histological and electrophysiological data from human cerebellar slices”*

*Beside that I would have following specific comments:*

We have revised the manuscript accordingly (see below).

*There are other approaches to Mean Field modeling than those based on Transfer Functions, some should be mentioned in the introduction. For example:*

*Schwalger, T., Deger, M., & Gerstner, W. (2017). Towards a theory of cortical columns: From spiking neurons to interacting neural populations of finite size. PLoS Computational Biology, 13(4), e1005507.*

*Coombes, S., & Byrne, Á. (2019). Next generation neural mass models. In Nonlinear Dynamics in Computational Neuroscience (pp. 1–16). Springer International Publishing.*

*Montbrió, E., Pazó, D., & Roxin, A. (2015). Macroscopic Description for Networks of Spiking Neurons. Physical Review X, 5(2), 021028.*

We have added to the introduction an overview of different MF approaches (Lorenzian functions and finite-element based) used in neuroscience to further clarify the context of the approach applied here:

*The mean-field (MF) approximation theory describes collective neural activities maintaining a direct link with the corresponding spiking neural network. The MF model formalism approximates high-dimensional random systems by averaging their original properties over degrees of freedom to compute the first two statistical moments of the system variables (e.g., mean and variance of the population firing rates in a neural network). Different mathematical approaches exist to derive such statistical descriptions depending on the complexity of the underlying network. While theoretical derivations in simpler settings allow to capture firing dynamics up to fast synchronized events (e.g., Lorentzian or finite-size effect-based formalisms) (10-13), the network considered here impedes such an approach. Instead, we use a formalism based on the definition of a transfer function (TF) that models the neuronal input-output relationship to investigate the properties of network responses to different input (7,10,11). The MF developed here is based on the TF approach that summarizes the neuronal and connectivity properties of an entire SNN through ad-hoc transfer functions (11–13) and captures the statistical properties of network activity by computing the probabilistic evolution of neuronal states at subsequent time intervals (10,14,15).*

We have further compared Transfer function-based formalism with Lorenzian functions in section 4.4 Limitations and future developments: *“The chosen mathematical formalism can also impact on the performance of the MF. MFs based on TFs are not intended to process frequency oscillations unlike MFs based on Lorentzian functions (101,102).”*

*The linked code is not a public repository*

A github repository will be available on request for the moment, and it will be publicly released after the acceptance of the paper. A first demo for MF prediction is already accessible by request on EBRAINS platform.

*The text needs to be checked for typos, for example:*

We went through the manuscript and fixed the typos.

*line 61 - behaviur;*

*line 482-Figure 3.5*

## Reviewer #3

*Lorenzi et al. present a study on the simulation of the cerebellar cortical neural circuit using the mean-field (MF) framework. While the MF method has potential for studying the cerebellar neural circuit, the authors have not made a strong case for publication. Overall, my rating of this paper is not favorable for publication or further review due to the lack of rigorous comparison between the reference and approximation, inconsistency with in vivo experimental data, and lack of testing with realistic stimuli.*
 *My first major concern is the lack of rigorous and quantitative comparison between the reference result (SNN) and the approximation.*

We appreciate the rigorous approach of this reviewer. Now, beyond descriptive statistics, a rigorous comparison is provided by computing the Root Mean Squared Error (RMSE) score. RMSE reflects the difference between having or not having spikes and we further discuss this aspect in section “MF tuning” clarifying: *“The cerebellar MF reproduced the known aspects of circuit physiology revealed experimentally. These include: the ability of GrCs both to respond to impulsive inputs with bursts curtailed by GoC inhibition and to faithfully follow slow input fluctuations (94); the ability of PCs to generate burst-pause responses accentuated by MLIs (48,51). The cerebellar MF behaved as a smoothing filter reproducing the trend of SNN simulations without capturing the intra-population variability (e.g., GrC fluctuations in response to a constant input - Figure 6A). Since the intra-population variability was not accounted for, the maximal GoC responses were underestimated by the MF, which approximated GoG to a homogeneous population neglecting their biological heterogeneity (88). The differences that could be observed between the MF and the corresponding SNN simulations could thus be explained as a drawback of the homogeneity assumption. Despite these aspects (further discussed in section 4.4) the MF predicted with good approximation the SNN cerebellar output, i.e., the PC activity (Boxplot in Figure 6).”* Additionally, we further deepen these concepts by adding a new section *4.4 Limitations and futute developments*

We have also quantified the correlation of population activity in cerebellar MF as well as in the cerebellar SNN computing Pearson Correlation Coefficients and we add Figure S1 reporting the correlation matrices. *Population activities predicted by cerebellar MF were highly cross-correlated, resulting in Pearson Correlation Coefficients always > 0.7 (Panel A). Correlation between population activities simulated by SNN is reported in Panel B; variability intrinsic in SNN led to lower correlations between pairs of population activities than in MF. These correlations matrices are reported for each input pattern. The input frequencies might be replaced with a probabilistic kernel to introduce variability in the MF formalism and reduce the inter-population correlations.*

Further, we have added a detailed study on emerging input-output (I-O) relationships across layers to investigate the functional regime of the model (*Figure 5) Cerebellar mean-field overall input/output relationship)*.

We hope that doubts are solved.

*For example, in Figure 3, the authors show transfer functions of neurons, but there is no quantification, only the best-fit parameters are shown.*

The semi-analytical representation of the transfer functions shown here comes out from the two-step fitting procedure as detailed in Zerlaut et al., 2018, which rely on a minimization algorithm, thus the results represent the optimal findings of this algorithm. Specifically, here the semi-analytical TFs are the output of two minimization algorithm with a tolerance of 10e-5. (i) Sequential Least Squares Programming algorithm and (ii) the Nelder-Mead was used to find out the optimal coefficient of the phenomenological threshold (scipy.optimize.minimize library).

*Similarly, in Figure 5, there is no quantification of the resemblance between SNN and MF simulation results.*

Former Figure 5 is now Figure 6 since we added the study of the emerging I-O relationships. We compute the RMSE and correlation matrices between SNN and MF simulations for each input present in Figure 5. Furthermore, In Figure 6 we add the indication of the standard deviation predicted by MF for each population (shade). As expected from the theoretical root of the population-based formalism and qualitatively confirmed by the plots in Figure 6 Panel A, the MF perform like a smoothing filter, showing a lower variability with respect to the SNN, which, indeed, is intended to include single-neuron specific features, resulting in a more heterogeneous representation. We further clarify this in the discussion: *“The cerebellar MF behaved as a smoothing filter reproducing the trend of SNN simulations without capturing the intra-population variability (e.g., GrC fluctuations in response to a constant input - Figure 6A).“*

Further, we computed the RMSE and discussed it in detail above.

*The authors claim similarity, but the blue curve undershoots the GoC PSTH, the orange curve overlays on top of the MLI PSTH, and the red curve does not capture the temporal dynamics of the GrC firing.*

We thank the reviewer for the comment. We have further explained the different nature of SNN and MF formalisms which are the reason for the differences pointed out by reviewer 3. SNN is a time-coded model in which the information is represented by the precise timing of spikes. On the other hand, MF is a rate-coded model which, instead of considering the precise timing of spikes, focuses on the overall activity level or the average firing rate of the neural populations. For this reason, the MF, as a different level of representation, cannot explain timing as the SNN but can translate some properties to the population level.

We have commented this in the discussion: “ *“The cerebellar MF reproduced the known aspects of circuit physiology revealed experimentally. These include: the ability of GrCs both to respond to impulsive inputs with bursts curtailed by GoC inhibition and to faithfully follow slow input fluctuations (94); the ability of PCs to generate burst-pause responses accentuated by MLIs (48,51). The cerebellar MF behaved as a smoothing filter reproducing the trend of SNN simulations without capturing the intra-population variability (e.g., GrC fluctuations in response to a constant input - Figure 6A). Since the intra-population variability was not accounted for, the maximal GoC responses were underestimated by the MF, which approximated GoG to a homogeneous population neglecting their biological heterogeneity (88). The differences that could be observed between the MF and the corresponding SNN simulations could thus be explained as a drawback of the homogeneity assumption. Despite these aspects (further discussed in section 4.4) the MF predicted with good approximation the SNN cerebellar output, i.e., the PC activity (Boxplot in Figure 6).”*”

We would like to further clarify this concept both in *MF tuning (section 4.1): “The differences that could be observed between the MF and the corresponding SNN simulations could thus be explained as a drawback of the homogeneity assumption”* and in section *4.4. Limitations and Future developments:* *” Specifically, translating the cerebellar SNN into the cerebellar MF involves simplifications (e.g., lack of plasticity rules) and assumptions (e.g., homogeneity inside the populations). In practice, this results in a cerebellar MF with the predictive capability of reproducing the effects of complex dynamics like burst-pause of PCs at a population level, but without modelling the neuronal coding over an heterogeneous neuronal population (103). For example, a cerebellar SNN was used to simulate the control of saccades movements exerted by two subpopulation of PCs (104). Conversely, the cerebellar MF assumes intra-population homogeneity and can’t be used to study such phenomena.”*

In section *4.3 Performance vs realism* we also clarify that MF and SNN could be used as complementary approach: “*“Theoretically, the cerebellar MF is not just a replacement for SNN, but rather a specific instrument for addressing questions at a mesoscale. SNNs and MFs indeed cover complementary aspects of brain function and dysfunction. For example, in Parkinson disease, a SNN was successfully used to simulate the thalamic and basal ganglia microcircuits in order to control deep brain stimulation electrodes (95,96). The SNN provided a description of microcircuit activity and of the involvement of dopaminergic neurons in tremor (97,98). On the other hand, MFs of the basal ganglia and thalamocortical circuit were used to embed neural mechanisms into the motor network involved in Parkinson disease and investigate large-scale oscillations (99). In dystonia, a cerebellar SNN embedding Dystonic alterations was successfully used to simulate alterations in PC firing rate , olivocerebellar connectivity, and parallel-fiber/PC spike-timing dependent plasticity (STDP) (100). While these microscopic properties may not be reproduced by the cerebellar MF, it may however reproduce the overall PC output alterations allowing to simulate the large-scale propagation of cerebellar abnormalities to other brain regions.”*”

and in section *4.4. Limitations and further developments* we provide a practical example of a case in which time-coding is essential to simulate a specific mechanism: “*For example, a cerebellar SNN was used to simulate the control of saccades movements exerted by two subpopulation of PCs (104). Conversely, the cerebellar MF assumes intra-population homogeneity and can’t be used to study such phenomena”*”

*On the right hand side, boxplots the mean firing rate (?) is shown, but again no statistical comparison either.*

The Boxplots of the average activity stimulated by SNN and MF are shown for Purkinje Cell to provide an immediate idea of the difference in data dispersion between SNN and MF; in spite of this theoretical difference, the MF predictions follow into the SNN variability. To quantify this, we added the scores explained in the answer above. *Second, the simulation results are inconsistent with in vivo experimental data. For instance, the modulation of the firing of the mossy fibers by various oscillations does not entrain the Purkinje cells, as shown in Ros et al., J Neurosci 2009. Additionally, the authors explore different synaptic parameters in Purkinje cells in Figure 6, but do not show heterogeneous responses that the Purkinje cells show potentially due to different excitation/inhibition balance in their inputs (Jelitai et al., Nat Comm, 2015). Particularly, the simulated Purkinje neurons never show the pausing response except for a unrealistic corner of their parameter space (Fig. 6B Top). This casts doubt on the biological relevance of their results.*

The MF is not intended to capture single-cell properties but rather an average population behavior, therefore, since not all PCs are physiologically generating a burst-pause response, it is possible that MF does not capture the heterogeneous response of a single or a small group of PCs. In section 2 Methods we clarify the terminology used through the manuscript: *“For the sake of simplicity, activity patterns in the MF are described using a terminology derived from the SNN, so that a rate-coded waveform with a peak followed by a reduction is called burst-pause to suggest that the generative mechanism is the same as that of a spike-coded pattern”*.

Further in section 4.4. Limitations and Future developments, we clarify these limitations by adding the references suggested by reviewer 3: “*Considering the regime of applicability, the formalism of our cerebellar MF is intended to work at an asynchronous state with irregular firing rate. Therefore, input oscillations are not processed by this formalism (Ros et al., J Neurosci 2009). Oscillations in Figure 6 are merely input-driven. To include frequency elaborations as intrinsic processes of the cerebellar MF itself, further improvements in the MF formalism should be introduced, e.g., embedding mechanisms to account for adaptation and neuronal heterogeneity (109). [ …] In practice, this results in a cerebellar MF with the predictive capability of reproducing the effects of complex dynamics like burst-pause of PCs at a population level, but without modelling the neuronal coding over an heterogeneous neuronal population (Jelitai et al., Nat Comm 2015)”*

It should be noted that the spiking neural networks used for reference (Geminiani et al., 2019) do not implement dendritic computation. This MF was developed using this state-of-art SNN in order to provide a bridge between micro and macroscale activity, translating biophysical single cell properties into average population activity. Therefore, for design choice, this MF couldn’t reproduce the phenomena related to a single cell dendritic signal processing and it is beyond the scope of the model presented here. Perhaps, this could be done in the future by using as reference a spiking neural network built up with multicompartmental neurons. An accurate evaluation of the tradeoff between using a multicompartmental neurons SNN and computational resources should be performed.

*Lastly, the MF simulation is tested only with limited or unrealistic protocols for external stimuli. For example, the authors did not test their models with mossy fiber bursts, which are commonly observed in vivo. Mossy fibers burst with the peak firing rate ~500 Hz with sensory stimuli (Rancz et al., Nature, 2007) and the similar phenomena are reported for the mossy fibers carrying motor information (Ohtsuka and Noda, Neurosci Res, 1992). The absence of this protocol limits the applicability of this study to real biological phenomena.*

We add a section (*4.4 Limits and future developments)* to explain the limitations of the cerebellar MF both from a theoretical point of view and in terms of detail. As explained above, the MF can’t reproduce the dendritic computation effects but it is intended to reproduce the effects of microstructural mechanisms.
